# Supplementary material for: Applicability of Oculomics for Individual Risk Prediction: Repeatability and Robustness of Retinal Fractal Dimension Using DART and AutoMorph
Source: Invest Ophthalmol Vis Sci. 2024 Jun 6;65(6):10. doi: 10.1167/iovs.65.6.10 (PMC11160956; doi:10.1167/iovs.65.6.10)

Supplementary

**Supplementary Figure 1:** Interchangeability of FD measurements by DART and AutoMorph. We use mean values per eye to reduce noise and exclude images with p(bad)>0.8. a) shows Bland-Altman plots using the original values for both Caledonia (i) and GRAPE (ii). b) shows Bland-Altman plots after DART FD values have been mapped to AutoMorph values using isotonic regression, to investigate whether it is possible to “translate” between the two tools. c) shows the isotonic regression lines and underlying data.

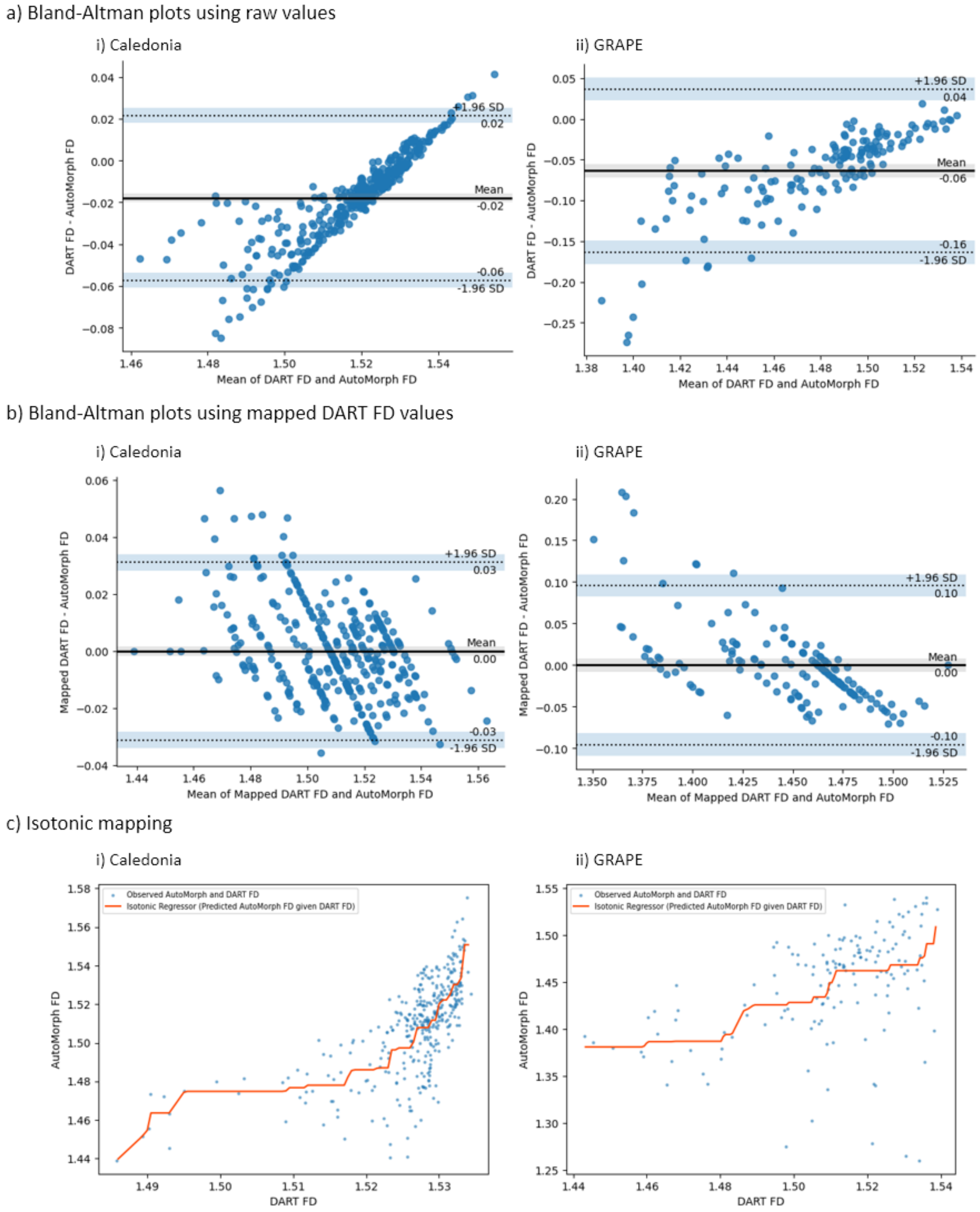

Supplement: Supplement 1 [file iovs-65-6-10_s001.pdf]
